# Protein Quality Control at the Mitochondrial Surface

**DOI:** 10.3389/fcell.2021.795685

**Published:** 2021-12-03

**Authors:** Fabian den Brave, Arushi Gupta, Thomas Becker

**Affiliations:** Institute of Biochemistry and Molecular Biology, Faculty of Medicine, University of Bonn, Bonn, Germany

**Keywords:** mitochondria, protein sorting, protein quality control, Cdc48, TOM complex

## Abstract

Mitochondria contain two membranes, the outer and inner membrane. The outer membrane fulfills crucial functions for the communication of mitochondria with the cellular environment like exchange of lipids via organelle contact sites, the transport of metabolites and the formation of a signaling platform in apoptosis and innate immunity. The translocase of the outer membrane (TOM complex) forms the entry gate for the vast majority of precursor proteins that are produced on cytosolic ribosomes. Surveillance of the functionality of outer membrane proteins is critical for mitochondrial functions and biogenesis. Quality control mechanisms remove defective and mistargeted proteins from the outer membrane as well as precursor proteins that clog the TOM complex. Selective degradation of single proteins is also an important mode to regulate mitochondrial dynamics and initiation of mitophagy pathways. Whereas inner mitochondrial compartments are equipped with specific proteases, the ubiquitin-proteasome system is a central player in protein surveillance on the mitochondrial surface. In this review, we summarize our current knowledge about the molecular mechanisms that govern quality control of proteins at the outer mitochondrial membrane.

## Introduction

Mitochondria are known as the powerhouse of the cell since they produce the bulk of energy for cellular processes. They also synthesize lipids and amino acids, form co-factors like iron-sulfur clusters and constitute a platform for cellular signaling in apoptosis and innate immunity (Spinelli and Haigis, 2018; Tiku et al., 2020). Mitochondria display specific features, which are due to their endosymbiotic origin. They contain their own genome, which encodes for eight proteins in the baker’s yeast *Saccharomyces cerevisiae* and 13 proteins in human mitochondria. The vast majority of about 1,000 mitochondrial proteins are produced as precursors on cytosolic ribosomes and imported into the target organelle. Mitochondria contain two membranes, the outer membrane and the inner membrane, which create two aqueous compartments, the intermembrane space and the matrix. The inner membrane forms large invaginations, termed cristae, which harbor the respiratory chain complexes. These membrane-integrated protein complexes transport electrons from reducing agents to oxygen to form water. The released energy is used to establish a proton gradient across the inner membrane that drives the F_1_F_O_-ATP synthase to produce ATP (von Ballmoos et al., 2009; Spinelli and Haigis, 2018; Kuhlbrandt, 2019).

The outer membrane constitutes the border of mitochondria to their cellular environment and is therefore critical for the integration of mitochondria within the cell. The outer membrane contains a few dozen integral membrane proteins (Schmitt et al., 2006; Zahedi et al., 2006; Morgenstern et al., 2017; Vögtle et al., 2017). In yeast, five proteins are embedded into the membrane by a β-barrel, whereas the majority is anchored via a single or several α-helical transmembrane segments. The voltage-dependent anion channel (VDAC or porin in yeast) mediates the exchange of small molecules and ions with the cellular environment (Colombini, 2012; Campo et al., 2017; Becker and Wagner, 2018). Protein translocases transport different types of precursor proteins that were produced on cytosolic ribosomes into and across the outer membrane (Dukanovic and Rapaport, 2011). Outer membrane proteins form contact sites to other cellular compartments. One example is the endoplasmic reticulum encounter structure (ERMES) in yeast that links mitochondria and the endoplasmic reticulum (ER) to facilitate lipid transfer (Kornmann et al., 2009; Ellenrieder et al., 2016). Finally, outer membrane localized proteins control fusion and fission of mitochondria (Escobar-Henriques and Anton, 2013; Kameoka et al., 2018; Tilokani et al., 2018).

Mitochondrial quality control mechanisms are critical to deal with the high load of incoming precursor proteins and to monitor their folding. The inner membrane and matrix are equipped with AAA proteases that remove misfolded or defective proteins: the *i*-AAA protease (Yme1/YME1L) of the inner membrane exposes its active site into the intermembrane space, the inner membrane embedded *m*-AAA protease (Yta10, Yta12/AFG3L2, paraplegin) exposes its catalytic center into the matrix and the soluble Pin1/LONP resides in the matrix (Gerdes et al., 2012; Deshwal et al., 2020; Song et al., 2021). The outer membrane lacks such specific AAA proteases. Instead, studies of the last few years uncovered that the ubiquitin proteasome system plays a central role in the degradation of aberrant proteins at the mitochondrial surface. We summarize here our current knowledge about surveillance of proteins on the mitochondrial surface.

## Overview of Protein Transport Into Mitochondria

Mitochondria have to import about 1,000 proteins in yeast and up to 1,500 proteins in humans that are produced on cytosolic ribosomes (Pagliarini et al., 2008; Morgenstern et al., 2017). Outer membrane proteins play a critical role in the import of precursor proteins into mitochondria (Endo and Yamano, 2009; Wiedemann and Pfanner, 2017; Hansen and Herrmann, 2019). The vast majority of mitochondrial proteins are synthesized by cytosolic ribosomes as precursors. Although co- and posttranslational import mechanisms have been reported, most mitochondrial precursor proteins are imported in a posttranslational manner (Becker et al., 2019; Bykov et al., 2020). Here, cytosolic chaperones like Hsp70 and Hsp90 guide these precursor proteins from ribosomes to the mitochondrial surface (Becker et al., 2019; Bykov et al., 2020). Receptors of the translocase of the outer membrane (TOM complex) recognize internal and cleavable mitochondrial targeting signals. The receptor protein Tom70 also binds to cytosolic Hsp70 and Hsp90 chaperones. The recruitment of chaperones to Tom70 is crucial to prevent proteotoxic stress due to aggregation of mitochondrial precursor proteins (Young et al., 2003; Backes et al., 2021). Alternatively, some hydrophobic precursor proteins are first guided to the ER and then transported to mitochondria (ER-SURF pathway) (Hansen et al., 2018). Here, the ER membrane serves as a scaffold to accommodate membrane proteins and thereby prevents their aggregation.

The TOM complex is the main entry gate for precursor proteins into mitochondria. The two peripheral TOM receptors, Tom20 and Tom70, bind to incoming precursor proteins. Proteins containing a cleavable presequence are primarily recognized by Tom20 (Abe et al., 2000; Yamano et al., 2008). Tom70 was previously thought to function as a receptor for integral membrane proteins, but several studies demonstrated that this receptor is also involved in the import of several presequence-containing proteins that contain internal mitochondrial targeting sequences (Hines et al., 1990; Brix et al., 1997; Young et al., 2003; Yamamoto et al., 2009; Backes et al., 2018; Backes et al., 2021). The central subunit of the TOM complex is the β-barrel protein Tom40, which forms the protein-conducting channel (Hill et al., 1998; Suzuki et al., 2004; Becker et al., 2005). High-resolution cryo-electron microscopic structures revealed that the TOM complex consists of two Tom40 lined by three small TOM subunits, Tom5, Tom6 and Tom7. Two Tom22 molecules link both Tom40-small Tom modules in the TOM complex. The overall dimeric structure is conserved from yeast to human (Bausewein et al., 2017; Araiso et al., 2019; Tucker and Park, 2019; Wang W. et al., 2020; Guan et al., 2021). Tom22 constitutes the docking site for Tom20 and Tom70 at the TOM complex to facilitate precursor transfer from the receptor proteins towards the translocation channel (van Wilpe et al., 1999).

**FIGURE 1:**
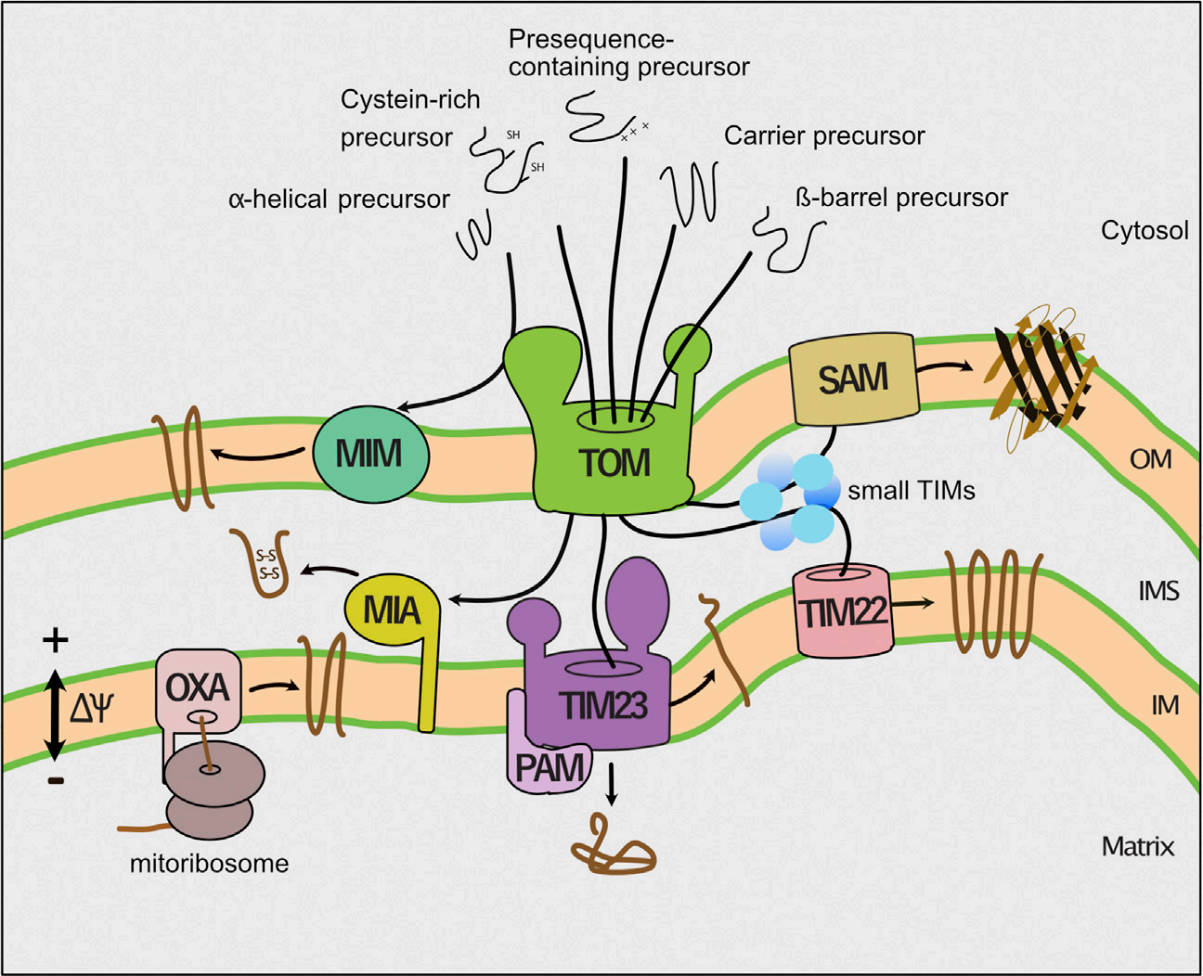
Pathways of mitochondrial protein import. Dedicated protein translocases import precursor proteins into the mitochondrial subcompartments. First, the translocase of the outer membrane (TOM complex) transports precursor proteins across the outer membrane (OM). Second, the presequence translocase (TIM23 complex) sort precursors with a cleavable presequence into the inner membrane (IM) and matrix. The presequence translocase-associated motor (PAM) cooperates with the TIM23 complex to complete protein translocation into the matrix. Third, the carrier translocase (TIM22) inserts hydrophobic multispanning proteins into the inner membrane that are guided by small TIM chaperones through the intermembrane space. The membrane potential (∆ψ) drives protein transport into the inner membrane and matrix. Fourth, the mitochondrial intermembrane space import and assembly (MIA) machinery mediates oxidative folding of proteins with a cysteine-rich targeting signal. Fifth, the sorting and assembly machinery (SAM complex) inserts β-barrel proteins into the outer membrane (OM). Sixth, the mitochondrial import (MIM) machinery integrates proteins with a single or multiple α-helical membrane spans into the outer membrane. Finally, the oxidase assembly (OXA) translocase inserts mitochondria-encoded proteins into the inner membrane.

After passage of the TOM channel, specific protein translocases sort the incoming precursor proteins to the mitochondrial subcompartments (Figure 1). The presequence translocase (TIM23 complex) transports precursor proteins into and across the inner membrane. The respiratory chain generates a membrane potential across the inner membrane that drives protein transport via the presequence pathways (Malhotra et al., 2013). Protein transport into the matrix additionally depends on the ATP consuming activity of the presequence translocase-associated motor (PAM). Multispanning inner membrane proteins like carriers lack a cleavable presequence. The small TIM chaperones of the intermembrane space transfer these hydrophobic proteins to a second protein translocase of the inner membrane, the carrier translocase (TIM22 complex). The TIM22 complex inserts carrier proteins in a membrane potential dependent manner into the inner membrane. The mitochondrial intermembrane space import and assembly (MIA) machinery imports cysteine-rich proteins such as the small TIM proteins into the intermembrane space and promotes their oxidative folding. Precursors of β-barrel proteins are first transported via the TOM complex across the outer membrane and subsequently guided by the small TIM chaperones to the sorting and assembly machinery (SAM complex), which integrates them into the outer membrane SAM complex. The mitochondrial import (MIM) machinery inserts proteins with an α-helical membrane anchor into the outer membrane. Remarkably, the majority of these precursor proteins is not transported via the TOM channel, but instead transferred to the MIM complex on the cytosolic side of the outer membrane. Finally, the oxidase assembly (OXA) translocase integrates mitochondria-encoded proteins and some nuclear encoded proteins into the inner membrane (Endo and Yamano, 2009; Jores and Rapaport, 2017; Wiedemann and Pfanner, 2017; Hansen and Herrmann, 2019; Drwesh and Rapaport, 2020).

## Defective Protein Import Into Mitochondria Causes Cellular Stress

### Cellular Stress Responses to Impaired Protein Import

Protein import into mitochondria is essential for cell viability and function. However, different scenarios could affect mitochondrial protein import. Precursor proteins have to be kept in a largely unfolded state to pass the Tom40 channel (Wiedemann et al., 2001; Shiota et al., 2015). Consequently, premature folding of precursor proteins blocks their passage of the TOM complex. If the N-terminal presequence of such prematurely folded precursor proteins already engages the presequence translocase of the inner membrane, the precursor protein will arrest within the protein translocon (Boos et al., 2019; Martensson et al., 2019). Particularly, precursor proteins that contain a bipartide targeting signal, which is composed of a mitochondrial presequence and an inner membrane sorting signal, are prone to clog the translocon (Weidberg and Amon, 2018). Moreover, mitochondrial dysfunction can affect the membrane potential across the inner membrane, which in turn impairs protein transport into mitochondria (Jin et al., 2010; Malhotra et al., 2013; Rolland et al., 2019).

Impaired protein transport into mitochondria causes cellular stress due to accumulation of mitochondrial precursor proteins (termed precursor overaccumulation stress (mPOS)) and induces a massive transcriptional reprogramming to deal with the proteotoxic stress (Wang and Chen, 2015; Boos et al., 2019). This stress response includes an increased expression and assembly of proteasomal subunits (termed the unfolded protein response activated by mistargeting of proteins (UPRam)) (Wrobel et al., 2015). As a result, the proteasomal activity in the cell is increased to degrade the precursor proteins. Precursor accumulation also induces the expression of chaperones and results in downregulation of general protein synthesis, including decreased expression of genes encoding for proteins involved in respiratory metabolism to reduce the protein load of the protein translocases (Wang and Chen, 2015; Boos et al., 2019). If the proteotoxic stress persists it will eventually cause cell death (Wang and Chen, 2015). The induced stress responses reveal that the maintenance of full protein import competence of mitochondria is critical to balance cellular proteostasis. Thus, molecular mechanisms are required that prevent clogging of the TOM channel to ensure proper protein import into mitochondria.

### Mislocalization of Mitochondrial Precursor Proteins

Non-imported mitochondrial precursor proteins can aggregate or mislocalize to different cellular compartments. Recent studies revealed that in yeast non-imported mitochondrial precursor proteins localize to the cytosol, the ER and the nucleus (den Brave et al., 2020; Doan et al., 2020; Shakya et al., 2021; Xiao et al., 2021). Several GFP-tagged and untagged matrix targeted precursor proteins have been found in the nucleus when protein import into mitochondria was impaired (den Brave et al., 2020; Shakya et al., 2021). The nucleus has a high concentration of proteasomes and is known to allow efficient degradation of aberrant proteins (Russell et al., 1999; Park et al., 2013; Enam et al., 2018; Frottin et al., 2019). Mitochondrial proteins accumulate in nuclear protein inclusions upon inhibition of proteasomal turnover (den Brave et al., 2020). Thus, transport to the nucleus could facilitate efficient degradation of non-imported mitochondrial precursor proteins. Outer and inner mitochondrial membrane proteins can mislocalize to the ER upon import failure (Doan et al., 2020; Shakya et al., 2021; Xiao et al., 2021). One possible reason is that the cytosolic protein targeting pathways to mitochondria and to the ER are closely linked to each other (Wang et al., 2010; Hansen et al., 2018; Becker et al., 2019; Bykov et al., 2020). For instance, some hydrophobic precursor proteins are transported via the ER surface to mitochondria (ER-SURF pathway; Hansen et al., 2018). The conserved P5A-ATPase (CATP-8 in *Caenorhabditis elegans*, Spf1 in yeast and ATP13A1 in humans) recognizes the transmembrane segment of mislocalized outer membrane proteins and dislocates them from the ER membrane (Krumpe et al., 2012; McKenna et al., 2020; Qin et al., 2020). In yeast, an interaction partner of Spf1, Ema19, binds to non-imported mitochondrial proteins and facilitates their degradation (Laborenz et al., 2021). Whether and how Ema19 cooperates with Spf1 remains to be investigated. Thus, non-imported mitochondrial precursor proteins are transported to different cellular compartments, where they challenge the compartment-specific quality control systems.

## Surveillance of Protein Entry in Mitochondria

### Cytosolic Quality Control of Mitochondrial Proteins

Protein quality control of mitochondrial precursor proteins starts already in the cytosol. The ubiquitin-proteasome system plays a central role in the removal of non-imported precursor proteins. Upon failure of protein import, the precursor proteins are ubiquitylated and delivered for proteasomal degradation (Radke et al., 2008; Bragoszewski et al., 2013; Habich et al., 2018; Kowalski et al., 2018; Mohanraj et al., 2019; Finger et al., 2020). Ubiquitylated proteins cannot pass the TOM channel to enter mitochondria (Kowalski et al., 2018). Ubiquitin is a highly conserved small protein of 76 amino acids that marks substrate proteins for degradation by the 26S proteasome (Amm et al., 2014; Ciechanover and Stanhill, 2014; Finley et al., 2016; Chen et al., 2021). Ubiquitylation of client proteins is mediated by an enzymatic cascade comprising an ubiquitin-activating enzyme (E1), an ubiquitin-conjugating enzyme (E2) and a ubiquitin ligase (E3) (Kerscher et al., 2006; Komander and Rape, 2012; Clague et al., 2015; Cappadocia and Lima, 2018). The substrate specificity in the ubiquitin pathway is largely determined by the large set of E3 ubiquitin ligases. There are up to 100 in yeast and several hundred E3 ubiquitin ligases in mammalian cells (Deshaies and Joazeiro, 2009; Chaugule and Walden, 2016; Zheng and Shabek, 2017; Walden and Rittinger, 2018). Subsequently, specific factors like ubiquilins in mammalian cells deliver ubiquitylated proteins for proteasomal degradation. Ubiquilins bind to the transmembrane domain of mitochondrial precursor proteins to prevent their aggregation (Itakura et al., 2016). Upon import failure, ubiquilins deliver the bound client proteins for proteasomal degradation (Itakura et al., 2016). Ubiquilins contain a ubiquitin-associated (UBA) domains, as well as a ubiquitin-like (UBL) domain. The UBA and UBL domains interact with each other. Prolonged binding of the precursor to ubiquilin due to impaired import results in ubiquitylation of the attached client protein. The ubiquitylated client protein is then bound by the UBA domain, disrupting its interaction to the UBL domain. The released UBL domain mediates docking to the proteasome to transfer the client protein for degradation (Elsasser et al., 2002; Funakoshi et al., 2002).

### Protein Quality Control at the Entry Gate of Mitochondria

**FIGURE 2:**
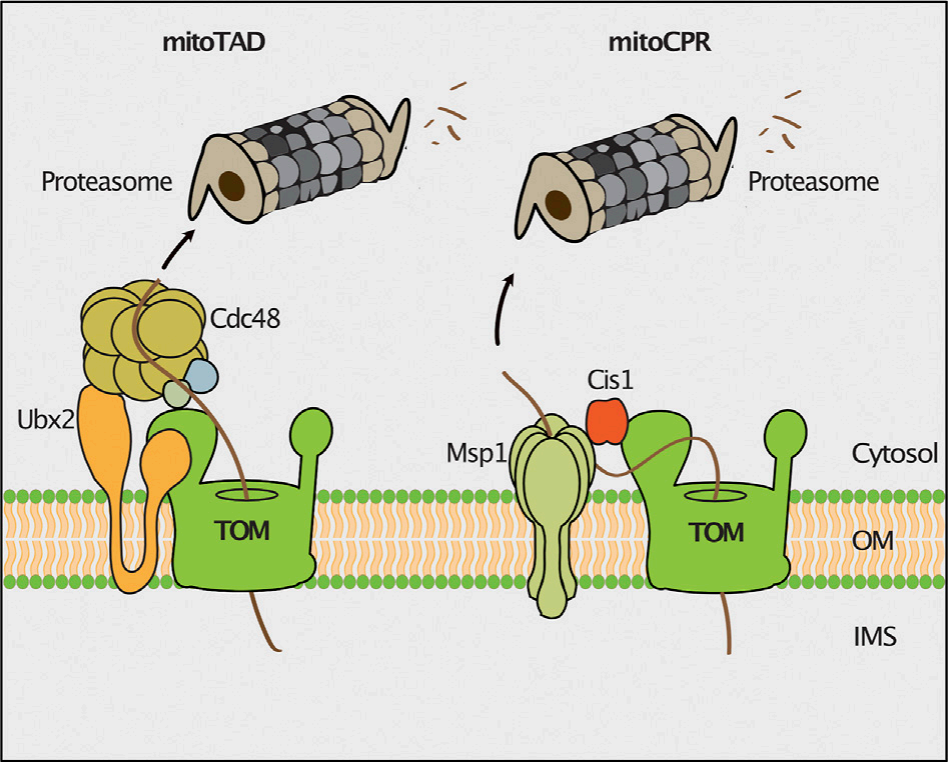
Quality control of protein import. The mitochondrial protein translocation-associated degradation (mitoTAD) pathway removes precursor proteins from the TOM complex that arrest in the translocon during transport. Ubx2 recruits the AAA-ATPase Cdc48 for extraction of stalled precursor proteins from the TOM channel for proteasomal turnover. In the mitochondrial compromised protein import response (mitoCPR) the expression of the *CIS1* gene is induced upon mitochondrial import stress. Cis1 links the membrane bound AAA-ATPase Msp1 to Tom70 to facilitate the extraction and subsequent proteasomal turnover of stalled precursor proteins.

Clogging of the TOM complex is deleterious for cell viability. Therefore, different molecular mechanisms operate at the translocase to remove precursor proteins that arrest during translocation in the TOM channel. The mitochondrial protein translocation-associated degradation (mitoTAD) pathway continuously monitors the TOM complex to prevent clogging of the translocation channel with precursor proteins (Figure 2; Martensson et al., 2019). A core subunit of this pathway is Ubx2, which was previously described to function in the endoplasmic reticulum associated degradation (ERAD) (Neuber et al., 2005; Schuberth and Buchberger, 2005). A fraction of Ubx2 is integrated into the mitochondrial outer membrane and binds to the TOM complex (Martensson et al., 2019). Ubx2 exposes a UBX domain, which is the docking site for the cytosolic AAA ATPase Cdc48 (p97/VCP in mammalian cells) at the TOM complex. Cdc48/p97 is a multifunctional protein that extracts proteins in different processes such as the ERAD, the ribosome-associated quality control (RQC) and the regulation of lipid droplet proteins (Olzmann et al., 2013; Franz et al., 2016; Wu and Rapoport, 2018; Joazeiro, 2019). Cdc48 harbors two ATPase domains and forms a hexameric structure with a central pore (DeLaBarre and Brunger, 2003). ATP hydrolysis drives the transport of the client protein through the central pore, thereby applying a pulling force on the substrate, which results in the extraction and unfolding of the bound protein (Bodnar and Rapoport, 2017). Cdc48 extracts translocation-arrested precursor proteins from the TOM channel and delivers them for proteasomal turnover (Martensson et al., 2019). The mechanism underlying substrate recognition and ubiquitylation in the mitoTAD pathway in yeast mitochondria is still unknown. In mammalian cells, the E3 ubiquitin ligase MARCH5 and the deubiquitylating enzyme USP30 have been shown to regulate the ubiquitylation of precursor proteins at the TOM complex (Ordureau et al., 2020; Phu et al., 2020). MARCH5-dependent ubiquitylation targets precursor proteins for degradation by the proteasome, whereas USP30 promotes import by removing ubiquitin from precursor proteins (Ordureau et al., 2020; Phu et al., 2020). It remains to be investigated whether also in mammalian cells the homologous proteins of Cdc48 (p97/VCP) and Ubx2 (UBXD8/FAF2) are involved in extraction of mitochondrial precursor proteins from the import channel.

The mitochondrial compromised protein import response (mitoCPR) is activated upon import failure in yeast cells (Figure 2). The accumulation of non-imported mitochondrial precursor proteins in the cell induces the expression of the cytosolic protein Cis1 (Weidberg and Amon, 2018; Boos et al., 2019). Cis1 in turn recruits the outer membrane bound AAA-ATPase Msp1 to the Tom70 receptor (Weidberg and Amon, 2018). Msp1 facilitates extraction of translocation-arrested precursor proteins from the TOM channel for their proteasomal degradation (Basch et al., 2020). How proteasomal targeting is achieved and whether Msp1 cooperates with components of the mitoTAD pathway in clearing proteins stalled in the TOM translocon, remains unclear.

**FIGURE 3:**
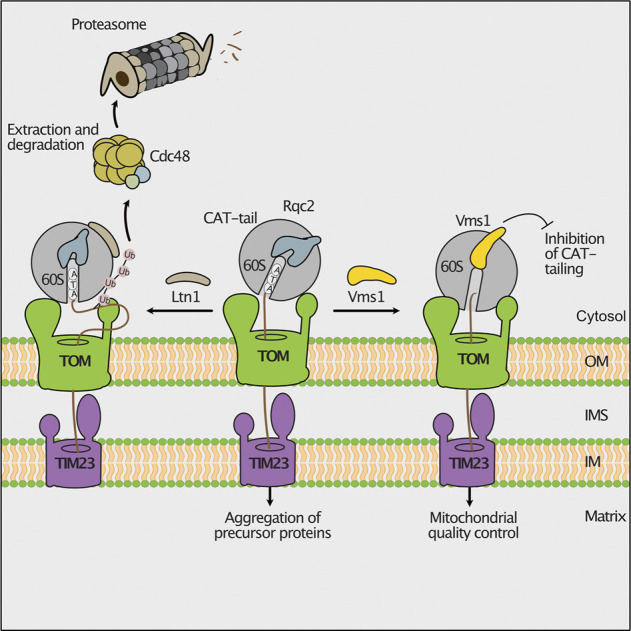
Ribosomal protein quality control during import. Stalling during translation activates the mitochondrial ribosome-associated protein quality control (RQC). Nascent chains can be ubiquitylated by the ribosome bound E3 ubiquitin-ligase Ltn1, allowing their degradation by cytosolic proteasomes **(left).** If the lysine residues of the stalled polypeptide are not accessible for ubiquitylation, the RQC component Rqc2 will add C-terminal alanine and threonine residues (CAT-tails) to the nascent chain. Imported CAT-tailed proteins form toxic aggregates in the mitochondrial matrix **(middle).** The peptidyl-tRNA hydrolase Vms1 releases the nascent polypeptide from the ribosome and blocks the Rqc2-mediated CAT-tailing. Upon import, these unmodified proteins are degraded by mitochondrial proteases **(right).**

Nascent mitochondrial precursor proteins can stall at the ribosome during translation. Possible reasons are the lack of a STOP codon, strong secondary structures in the mRNA or deficiencies in the amounts of some amino acids or tRNAs (Brandman and Hegde, 2016). The ribosome-associated quality control (RQC) delivers such stalled nascent polypeptides to proteasomes for degradation (Joazeiro, 2019). In this pathway, the ribosomal subunits dissociate and the nascent protein remains bound to the 60S subunit. Subsequently, the E3 ubiquitin ligase Ltn1 ubiquitylates nascent polypeptides to mark them for proteasomal turnover (Bengtson and Joazeiro, 2010; Brandman et al., 2012). In case no lysine is accessible for ubiquitylation since they are buried in the ribosomal exit tunnel, Rqc2 binds to ribosomes and adds C-terminally alanine and threonine residues (CAT-tail) to the nascent chain. Thereby, additional lysine residues may exit the ribosomal tunnel and be ubiquitylated by Ltn1 (Shen et al., 2015). During co-translational protein import into mitochondria, the nascent chain is directly transferred from the ribosome to the TOM channel. Consequently, lysine residues can escape ubiquitylation by Ltn1 although CAT-tailing occurs. Such CAT-tailed proteins can be imported into mitochondria, where they form toxic inclusions (Figure 3; Izawa et al., 2017). This detrimental import of CAT-tailed proteins is prevented by the conserved protein Vms1. Vms1 like its human homolog ANKZF1 functions as peptidyl-tRNA hydrolase and releases the nascent chain from the 60S ribosomal subunit (Izawa et al., 2017; Verma et al., 2018; Zurita Rendon et al., 2018). Furthermore, Vms1 hinders Rqc2 from binding to ribosomes and therefore prevents CAT-tailing (Izawa et al., 2017). The released nascent polypeptides are imported into mitochondria and degraded by mitochondrial proteases (Figure 3; Izawa et al., 2017).

## Removal of Mislocalized Proteins From the Outer Membrane

**FIGURE 4:**
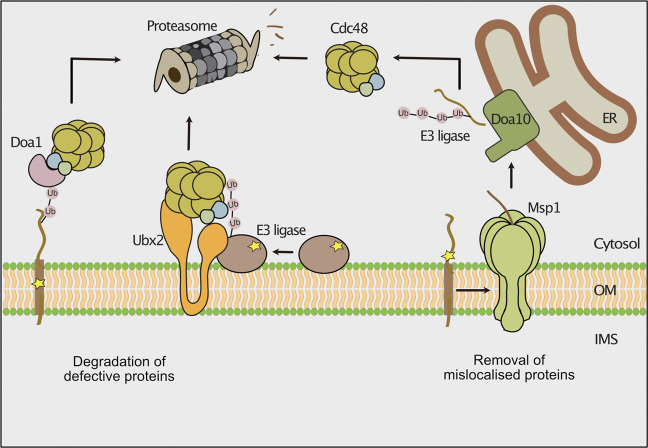
Degradation pathways of outer membrane proteins. The cytosolic ubiquitin-proteasome system removes misfolded or mislocalized proteins from the outer mitochondrial membrane. Defective outer membrane proteins are ubiquitylated by different E3 ubiquitin ligases. The AAA-ATPase Cdc48 is recruited to the outer membrane by its ubiquitin binding co-factors Doa1 and Ubx2 and powers the extraction of substrate proteins for proteasomal turnover. The AAA-ATPase Msp1 extracts mislocalized tail-anchored proteins from the outer membrane. The ER localized E3 ubiquitin ligase Doa10 ubiquitylates these proteins and Cdc48 delivers them for proteasomal degradation.

An intertwined cytosolic chaperone network guides membrane proteins to the outer mitochondrial membrane, ER and peroxisomes (Guna and Hegde, 2018; Becker et al., 2019; Deuerling et al., 2019; Walter and Erdmann, 2019; Bykov et al., 2020). Defects in the targeting pathways can lead to mislocalization of ER- or peroxisome resident proteins to the outer mitochondrial membrane (Schuldiner et al., 2008; Jonikas et al., 2009; Chen et al., 2014; Okreglak and Walter, 2014; Vitali et al., 2018). To avoid overloading with mistargeted proteins, clearance pathways exist to recognize and remove such proteins. In the mitochondrial outer membrane, mistargeted proteins with a C-terminal membrane anchor (C-tail anchored) are removed by the conserved AAA ATPase Msp1 (ATAD1 in mammals) (Figure 4) that also functions in the mitoCPR pathway as described above (Chen et al., 2014; Okreglak and Walter, 2014; Wohlever et al., 2017; Weidberg and Amon, 2018). The protein has a dual localization to mitochondria and peroxisomes (Weir et al., 2017). Msp1 is N-terminally anchored to the membrane, exposing a single ATPase domain and forms a hexameric complex with a central pore (Wohlever et al., 2017; Castanzo et al., 2020; Wang et al., 2020). The cytosolic exposed AAA domain drives in an ATP-dependent manner the client protein through the central pore (Castanzo et al., 2020; Wang L. et al., 2020). Different modes of substrate binding by Msp1 have been reported (Wang and Walter, 2020; Dederer and Lemberg, 2021). The transmembrane domain of mislocalized proteins do not perfectly match the dimension of the outer membrane lipid bilayer, leading to exposure of hydrophobic domains, which are bound by Msp1 (Li et al., 2019; Castanzo et al., 2020; Wang L. et al., 2020). Furthermore, unassembled proteins expose regions that are normally covered by a partner protein. In this case, Msp1 recognizes such regions and dislocates the orphaned proteins (Weir et al., 2017; Dederer et al., 2019). Following extraction by Msp1 the tail-anchored proteins are first transferred to the ER, where they are ubiquitylated by the membrane bound E3-ubiquitin ligase Doa10 (Dederer et al., 2019; Matsumoto et al., 2019). Subsequently, Cdc48 delivers these proteins for proteasomal degradation (Matsumoto et al., 2019). It is unclear why such proteins are not directly targeted from the mitochondrial surface to the proteasome for degradation. However, this mechanism might provide a second chance for mistargeted proteins to reach their correct destination.

## Quality Control of Misfolded Outer Membrane Proteins

The ubiquitin-proteasome system removes aberrant or unassembled proteins form the outer membrane of mitochondria under constitutive or stress conditions (Taylor and Rutter, 2011; Bragoszewski et al., 2017; Ravanelli et al., 2020; Ruan et al., 2020; Song et al., 2021). Furthermore, selective removal of proteins is an important mode to regulate different mitochondrial processes like mitophagy, apoptosis and mitochondrial dynamics (Youle, 2019; Deshwal et al., 2020; Escobar-Henriques and Anton, 2020; Song et al., 2021). In analogy to the ERAD, the pathway for degrading mitochondrial outer membrane proteins by the proteasome was termed mitochondria-associated degradation (MAD). Degradation pathways of a few peripheral membrane proteins and proteins with α-helical membrane spans have been described (Wu et al., 2016; Chowdhury et al., 2018; Wu et al., 2018; Goodrum et al., 2019; Metzger et al., 2020; Nahar et al., 2020). How defective β-barrel proteins are removed from the outer membrane remains to be discovered.

A set of cytosolic E3 ubiquitin ligases has been reported to ubiquitylate outer membrane proteins. In yeast, the E3 ubiquitin ligases Rsp5 and Mdm30 mediate ubiquitylation of components of ER-mitochondria contact sites and the GTPase Fzo1 (fuzzy onion) that mediates mitochondrial fusion (Cohen et al., 2008; Wu et al., 2016; Belgareh-Touze et al., 2017; Goodrum et al., 2019). Mdm30 belongs to the F-box proteins, which determine substrate specificity of SCF (Skp, Cullin, F-box) ubiquitin ligase complexes (Skaar et al., 2013). The E3 ubiquitin ligase Rsp5 fulfills several functions in the cell such as regulation of the biosynthesis of unsaturated fatty acids, multivesicular body formation and protein degradation upon heat stress (Hoppe et al., 2000; Oestreich et al., 2007; Fang et al., 2014; Li et al., 2015). The proteasomal turnover of the mutant variants of Sam35 and Sen2 was shown to depend on the E3 ubiquitin ligases San1 and Ubr1 (Figure 4; Metzger et al., 2020). Both E3 ligases mediate the turnover of misfolded proteins from various cellular compartments including the nucleus, the ER, cytosol and mitochondria (Gardner et al., 2005; Heck et al., 2010; Nillegoda et al., 2010; Prasad et al., 2018; Szoradi et al., 2018; Shakya et al., 2021). Future work has to define how these E3 ubiquitin ligases are recruited to the outer membrane.

The removal of damaged outer membrane proteins by the proteasome requires a preceding membrane extraction step (Figure 4). Cdc48 in conjunction with multiple co-factors has been shown to mediate turnover of outer membrane proteins (Heo et al., 2010; Wu et al., 2016; Metzger et al., 2020). Different co-factors recruit Cdc48 to mitochondria. First, Ubx2 mediates the Cdc48-dependent turnover of peripheral proteins and of the integral membrane protein Fzo1 (Chowdhury et al., 2018; Metzger et al., 2020; Nahar et al., 2020). Second, Doa1 binds to Cdc48 in complex with Ufd1 and Npl4 (Rumpf and Jentsch, 2006). Several studies showed that Doa1 is involved in the turnover of mitochondrial outer membrane proteins (Wu et al., 2016; Goodrum et al., 2019; Metzger et al., 2020). Finally, Vms1 has a binding domain for Cdc48 and localizes to the mitochondrial outer membrane upon oxidative stress (Heo et al., 2010). The binding of Vms1 to mitochondria depends on oxidized ergosterol in the outer membrane and does not require additional interactions with membrane bound proteins (Nielson et al., 2017). The role of Vms1 in the degradation of outer membrane proteins is currently unclear. While it was initially reported that Vms1 targets Fzo1 for degradation (Heo et al., 2010), later studies found the degradation of Fzo1 to be independent of Vms1 (Esaki and Ogura, 2012; Wu et al., 2016).

It was reported that outer membrane proteins like Om45 and Tom22 that expose a soluble domain towards the intermembrane space can be degraded by the hexameric AAA-protease Yme1 (Wu et al., 2018). Yme1 is a multifunctional protease conserved from yeast to human that exposes its catalytic domain into the intermembrane space. It regulates mitochondrial fusion, lipid trafficking and protein transport to adjust mitochondrial biogenesis and function to cellular requirements (Baker et al., 2012; Schreiner et al., 2012; Rainbolt et al., 2013; Anand et al., 2014; MacVicar et al., 2019). Whether the ubiquitin-proteasome system is also involved in the degradation of these outer membrane proteins remains unclear. A recent study revealed that Yme1 and the ubiquitin-proteasome system can cooperate in protein degradation. The inner membrane-anchored NADH dehydrogenase Nde1 exists in two topomers. One topomer exposes the catalytic center into the intermembrane space, while the other topomer spans the outer membrane and is exposed to the cytosol. The ubiquitin-proteasome system degrades the cytosolic form, while the remaining part of the protein is removed by Yme1 (Saladi et al., 2020). Respiratory deficient cells accumulate the cytosol-exposed form of Nde1 that initiates cell death (Saladi et al., 2020).

## Selective Degradation of Outer Membrane Proteins

### Quality Control Regulates Mitochondrial Morphology

**FIGURE 5:**
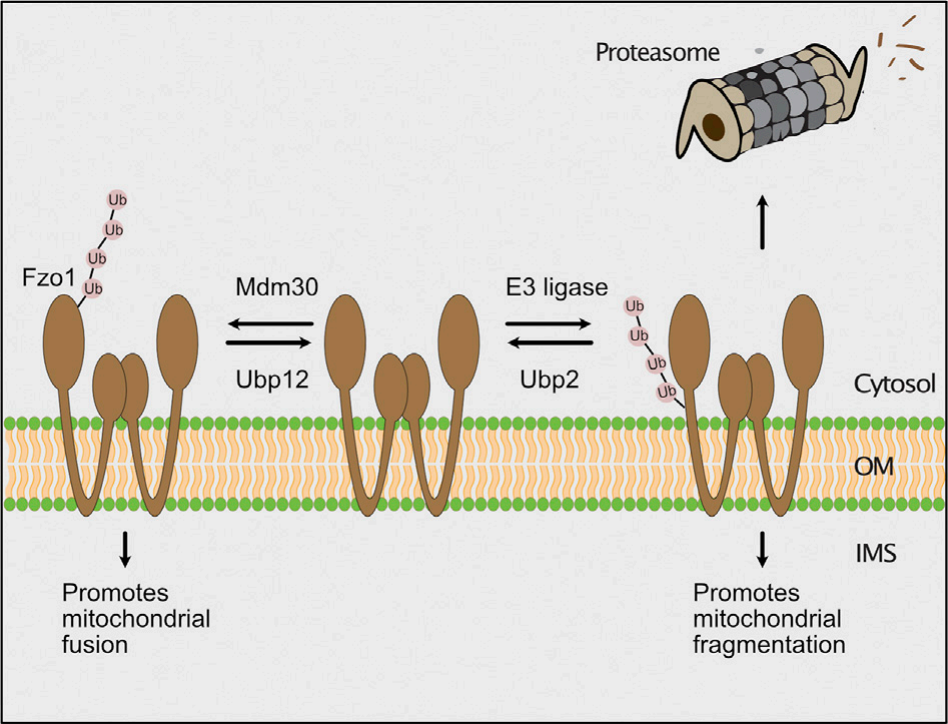
The ubiquitin-proteasome system regulates mitochondrial fusion. In yeast, the GTPase Fzo1 (fuzzy onion) promotes fusion of mitochondria. The F-box protein Mdm30 mediates ubiquitylation of Fzo1 at specific lysines to stimulate mitochondrial fusion. Fusion-promoting ubiquitylation is reversed by the deubiquitylating enzyme Ubp12. Alternatively, an unknown E3 ubiquitin ligase ubiquitylates Fzo1 on different lysine residues, which leads to its proteasomal turnover. The degradation of Fzo1 inhibits fusion and results in mitochondrial fragmentation due to ongoing fission. Proteolytic ubiquitylation of Fzo1 is inhibited by the deubiquitylating enzyme Ubp2.

The role of ubiquitylation and proteasomal turnover on the regulation of mitochondrial dynamics have been extensively studied. Mitochondria form a tubular network, which is constantly remodeled by opposing fusion and fission events. Both processes are mediated by large dynamin-like GTPases mediating outer membrane fusion (Fzo1 in yeast, mitofusins Mfn1/Mfn2 in mammals), inner membrane fusion (Mgm1 in yeast, Opa1 in mammals) and mitochondrial fission (Dnm1 in yeast, Drp1 in mammals) (Bleazard et al., 1999; Escobar-Henriques and Anton, 2013; Tilokani et al., 2018; Murata et al., 2020; Yapa et al., 2021). In particular turnover of Fzo1/mitofusins is a common mechanism to block fusion, thereby achieving mitochondrial fragmentation due to ongoing fission (Neutzner et al., 2007; Tanaka et al., 2010; Anton et al., 2013; Kim et al., 2013; Zhang et al., 2017; Goodrum et al., 2019). In yeast, the function of Fzo1 is fine-tuned by ubiquitylation and deubiquitylation (Figure 5; Cohen et al., 2008; Anton et al., 2013). Ubiquitylation of Fzo1 at specific lysine residues by Mdm30 promotes mitochondrial fusion (Fritz et al., 2003; Escobar-Henriques et al., 2006; Cohen et al., 2008; Anton et al., 2013), whereas ubiquitylation of alternative lysine residues of Fzo1 increases its degradation (Anton et al., 2013). The cytosolic deubiquitylase Ubp12 antagonizes Mdm30-mediated ubiquitylation and thereby inhibits mitochondrial fusion. Conversely, Ubp2 supports mitochondrial fusion by removing ubiquitylated species, which promote proteasomal turnover (Anton et al., 2013). In this process, Cdc48 functions as a regulatory hub, which is recruited by Fzo1 ubiquitylation and binds the deubiquitylating enzymes acting on Fzo1 (Simoes et al., 2018; Anton et al., 2019; Schuster et al., 2020). In contrast to its role in outer membrane quality control, Cdc48 appears to promote mitochondrial fusion by stabilizing ubiquitylated Fzo1 under non-stressed conditions (Simoes et al., 2018). Similarly, mammalian mitofusins are ubiquitylated by several E3 ubiquitin ligases (MARCH5, HUWE1, Parkin, Gp78), which regulate their degradation and function in fusion (Gegg et al., 2010; Tanaka et al., 2010; Leboucher et al., 2012; Fu et al., 2013; Yun et al., 2014; Tang et al., 2015; Mukherjee and Chakrabarti, 2016).

### Regulation of Mitophagy by the Ubiquitin Proteosome System

The activity of the ubiquitin proteasome system on mitochondrial outer membrane proteins is linked to mitophagy in mammalian cells. Mitophagy is the removal of damaged mitochondria by selective autophagy (Harper et al., 2018; Pickles et al., 2018; Onishi et al., 2021). Central players of this pathway are the PTEN-induced kinase 1 (PINK1) and the E3 ubiquitin ligase Parkin. In healthy mitochondria, PINK1 is imported into mitochondria and processed by the mitochondrial processing peptidase and the inner membrane rhomboid protease PARL (Jin et al., 2010). The remaining soluble fragment retro-translocates into the cytosol, where it is degraded by the proteasome (Yamano and Youle, 2013). Upon mitochondrial dysfunction, the reduced membrane potential causes stalling of PINK1 at the TOM complex (Lazarou et al., 2012). PINK1 phosphorylates a second PINK1 protein, ubiquitin and Parkin, which results in recruitment and activation of Parkin (Geisler et al., 2010; Matsuda et al., 2010; Narendra et al., 2010; Ziviani et al., 2010; Okatsu et al., 2012; Shiba-Fukushima et al., 2014; Wauer et al., 2015; Rasool et al., 2018). Parkin in turn ubiquitylates several proteins at the outer membrane including the mitofusins (Gegg et al., 2010; Poole et al., 2010; Tanaka et al., 2010; Ziviani et al., 2010; Wang et al., 2011). The human homolog of Cdc48, p97/VCP extracts ubiquitylated mitofusins from the outer membrane to target them for proteasomal turnover (Tanaka et al., 2010; McLelland et al., 2018). Thereby, fusion of the damaged mitochondria is blocked to facilitate their removal by mitophagy. Moreover, ubiquitylated outer membrane proteins serve as a docking site for ubiquitin-binding autophagy receptors like p62, which initiate further steps in mitophagy (Geisler et al., 2010; Lazarou et al., 2015; Harper et al., 2018; Pickles et al., 2018; Onishi et al., 2021).

## Mitochondrial-Derived Vesicles

An alternative process for the degradation of mitochondrial proteins is the formation of mitochondrial-derived vesicles (MDVs) in mammalian cells or mitochondrial-derived compartments (MDCs) in yeast (Suguira et al., 2014). Yeast MDCs contain only a small subset of mitochondrial proteins and show enrichment of the outer membrane protein Tom70. The separation of MDCs from mitochondria requires the dynamin-like GTPase Dnm1 (Hughes et al., 2016). The formation of MDCs was shown to maintain mitochondrial function during ageing in yeast (Hughes et al., 2016). Moreover, MDCs are formed upon toxic increase of amino acid levels, where they function in segregating amino acid carriers from the mitochondrial surface (Schuler et al., 2021). Different sub-populations of mammalian MDVs have been described containing the outer membrane or both mitochondrial membranes (Soubannier et al., 2012). They deliver cargo proteins to peroxisomes and multivesicular bodies/lysosomes for degradation as well as to mediate *de novo* biogenesis of peroxisomes (Neuspiel et al., 2008; Soubannier et al., 2012; Suguira et al., 2017). The formation of MDVs occurs under basal growth conditions but is also triggered by cellular stress conditions such as oxidative stress or hypoxia (McLelland et al., 2014; Cadete et al., 2016; Matheoud et al., 2016; Li et al., 2020). In particular, MDVs function in targeting mitochondrial antigens for presentation by major histocompatibility complex class I (MHCI) on the cell surface in immune signaling (Matheoud et al., 2016). MDV-dependent antigen presentation is inhibited by PINK1 and Parkin (Matheoud et al., 2016), which however promote MDV formation under oxidative stress conditions (McLelland et al., 2014). MDCs and MDVs provide a safety mechanism that might be particularly important to rescue more bulky defects of mitochondria.

## Conclusion

Protein quality control at the mitochondrial surface is essential for cell viability. Defects in quality control result in accumulation and mislocalization of mitochondrial proteins, which causes proteotoxic stress and eventually cell death. The ubiquitin-proteasome system monitors outer membrane proteins and protein import into mitochondria under constitutive and stress conditions. Two AAA ATPases, Cdc48 and Msp1, extract translocation-stalled precursor proteins from the TOM channel and aberrant or mislocalized proteins from the outer membrane for proteasomal turnover. Although several factors of these processes have been identified, our knowledge about key steps of these mechanisms such as substrate recognition and ubiquitylation are limited. Furthermore, the clearance mechanisms have only been described for a limited subset of proteins. It remains unknown whether the mitoTAD pathway specifically removes presequence-containing proteins or also other mitochondrial proteins that arrest in the TOM complex during translocation. While the removal of peripheral membrane proteins and proteins with an α-helical membrane anchor have been characterized, the degradation of damaged β-barrel proteins or protein complexes remains to be clarified. Strikingly, degradation of mislocalized proteins from the outer membrane involves the cooperation of mitochondria-associated, cytosolic and ER-localized factors (Dederer et al., 2019; Matsumoto et al., 2019). Further studies have to reveal how the interplay of the different components is coordinated across cellular compartments. Overall, the quality control mechanisms on the mitochondrial surface are an emerging field with many exciting discoveries within the past few years. Understanding these mechanisms will be crucial to understand how mitochondria are integrated into the cellular environment.
